# MethGET: web-based bioinformatics software for correlating genome-wide DNA methylation and gene expression

**DOI:** 10.1186/s12864-020-6722-x

**Published:** 2020-05-29

**Authors:** Chin-Sheng Teng, Bing-Heng Wu, Ming-Ren Yen, Pao-Yang Chen

**Affiliations:** 1 Institute of Plant and Microbial Biology, Academia Sinica, No. 128, Section 2, Academia Rd, Nangang District, Taipei City, 11529 Taiwan; 2 Department of Agronomy, National Taiwan University, Taipei, 10617 Taiwan; 3 Department of Industrial Management, National Taiwan University of Science and Technology, Taipei, 10607 Taiwan

**Keywords:** DNA methylation, Gene expression, Epigenome, Correlation, Bioinformatics, Next-generation sequencing, Web server

## Abstract

**Background:**

DNA methylation is a major epigenetic modification involved in regulating gene expression. The effects of DNA methylation on gene expression differ by genomic location and vary across kingdoms, species and environmental conditions. To identify the functional regulatory roles of DNA methylation, the correlation between DNA methylation changes and alterations in gene expression is crucial. With the advance of next-generation sequencing, genome-wide methylation and gene expression profiling have become feasible. Current bioinformatics tools for investigating such correlation are designed to the assessment of DNA methylation at CG sites. The correlation of non-CG methylation and gene expression is very limited. Some bioinformatics databases allow correlation analysis, but they are limited to specific genomes such as that of humans and do not allow user-provided data.

**Results:**

Here, we developed a bioinformatics web tool, MethGET (Methylation and Gene Expression Teller), that is specialized to analyse the association between genome-wide DNA methylation and gene expression. MethGET is the first web tool to which users can supply their own data from any genome. It is also the tool that correlates gene expression with CG, CHG, and CHH methylation based on whole-genome bisulfite sequencing data. MethGET not only reveals the correlation within an individual sample (single-methylome) but also performs comparisons between two groups of samples (multiple-methylomes). For single-methylome analyses, MethGET provides Pearson correlations and ordinal associations to investigate the relationship between DNA methylation and gene expression. It also groups genes with different gene expression levels to view the methylation distribution at specific genomic regions. Multiple-methylome analyses include comparative analyses and heatmap representations between two groups. These functions enable the detailed investigation of the role of DNA methylation in gene regulation. Additionally, we applied MethGET to rice regeneration data and discovered that CHH methylation in the gene body region may play a role in the tissue culture process, which demonstrates the capability of MethGET for use in epigenomic research.

**Conclusions:**

MethGET is a Python software that correlates DNA methylation and gene expression. Its web interface is publicly available at https://paoyang.ipmb.sinica.edu.tw/Software.html. The stand-alone version and source codes are available on GitHub at https://github.com/Jason-Teng/MethGET.

## Background

Epigenetics is the study of heritable changes in gene expression that do not involve changes in DNA sequences [1]. DNA methylation is one of the best-studied epigenetic mechanisms and refers to a process by which a methyl group is added to a cytosine [2]. In plants, DNA methylation is found in three sequence contexts: CG, CHG, and CHH (H represents A, T or C), whereas in animals, it is mostly observed at CG sites [3]. CG, CHG and CHH methylation is established and maintained by different methyltransferases to achieve different biological outcomes, such as the silencing of transposable elements [4], genomic imprinting [5], and, most importantly, gene regulation [6]. DNA methylation at different genomic locations may have different impacts on regulating the expression of genes and transposable elements (TEs) [7]. Typically, DNA methylation in the promoter region may repress gene expression [8]. In the gene body, CG methylation is weakly positively correlated with gene expression in humans, while in Arabidopsis, modest CG methylation is related to higher gene expression [9, 10]. Although the global trends of the correlation described above have been reported, variability exists for individual genes, and more recent research has shown that the correlation between promoter methylation and gene expression is not always negative [11–13].

Dynamic changes in DNA methylation in the genome-wide profile (i.e., methylome) often affect gene expression with specific functional outcomes [14]. For instance, methylation changes play a role in gene regulation during sexual reproduction in both plants and animals [15]. In plants, DNA methylation can shape the transcriptome of the plant during seed germination and under biotic and abiotic stresses [15, 16]. In mammals, alterations of DNA methylation have been shown to be associated with altered gene expression in the development of cancer and cardiovascular diseases [17]. The relationship between methylation changes and gene expression changes under different biological conditions and at different timepoints is important, but the effects of DNA methylation on gene expression remain unclear and complicated [18]. Therefore, the measurement of their correlation is of significance to aid in the understanding of epigenetic regulatory networks.

Whole-genome bisulfite sequencing (WGBS) enables genome-wide analyses of cytosine methylation at single-nucleotide resolution [19], whereas RNA-sequencing (RNA-seq) can quantify gene expression by counting the reads mapped to the transcriptome [20]. There are several bioinformatics tools for DNA methylation analyses, but only a few can correlate DNA methylation and gene expression for customized analyses, such as COHCAP [21], PiiL [22], and ViewBS [23]. COHCAP and PiiL can integrate DNA methylation with gene expression, but they are restricted to CpG methylation analyses. ViewBS can correlate between non-CG methylation and gene expression, but the users need to process the data first to allow correlation analyses. MethHC [24] and iMETHYL [25] are databases of methylation and gene expression. They do not allow users to provide their own data, and they can only be applied to specific species. Therefore, bioinformatics tools specialized for evaluating the correlation between DNA methylation and gene expression could help facilitate epigenomic research.

In this research, we developed MethGET, web-based bioinformatics software for analyzing the correlation between genome-wide DNA methylation and gene expression. MethGET allows users to upload their own DNA methylation and gene expression data for any species. MethGET includes single-methylome analyses for viewing the correlation within a single sample and multiple-methylome analyses for detecting the correlations between DNA methylation changes and gene expression changes between two groups of samples. It also determines DNA methylation in different contexts (CG, CHG, and CHH) and across different genomic regions (gene body, promoter, exon, and intron) to explore the different roles of methylation mechanisms in gene expression. We demonstrated the capability of MethGET with Japonica rice data, and MethGET revealed a decrease in both CHH methylation and gene expression in most genes in the gene body region as the embryo developed into a regenerated callus, which was not reported in the original paper [26] and warrants further investigation. Thus, MethGET serves as a useful tool for scientists to unveil the role of DNA methylation in regulating gene expression.

## Methods

**Fig. 1:**
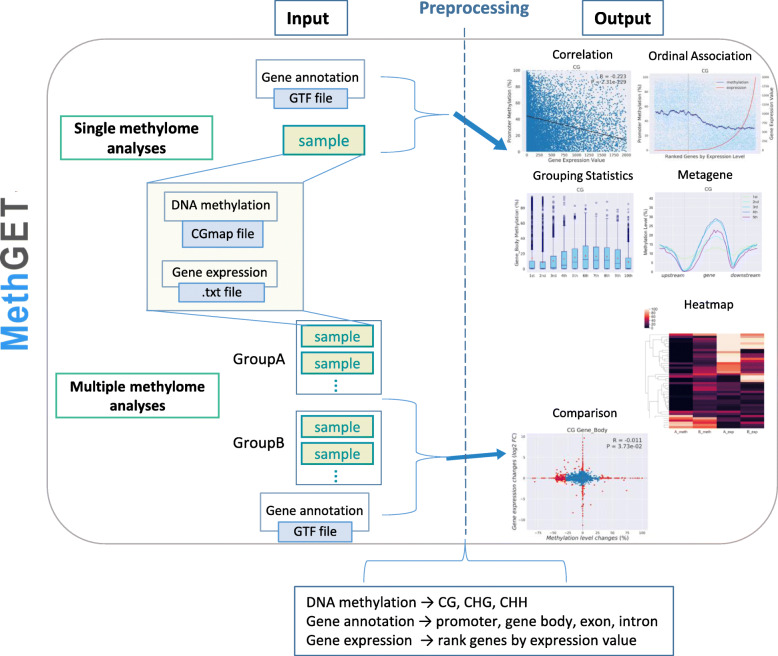
Schematic diagram of MethGET. The diagram shows the inputs and outputs of single-methylome analyses and multiple-methylome analyses

MethGET is a Python software that performs various analyses, including single-methylome analyses and multiple-methylome analyses (Fig. 1). MethGET uses DNA methylation, gene expression, and gene annotation data as the input for data preprocessing. In single-methylome analyses, the correlations within a single sample are detected; these analyses include the following: 1) correlation analyses of genome-wide DNA methylation and gene expression (correlation); 2) ordinal association analyses with genes ranked by gene expression level (ordinal association); 3) distribution of DNA methylation by groups of genes with different expression levels (grouping statistics); and 4) average methylation level profiling according to different expression groups around genes (metagene). In multiple-methylome analyses, two groups of samples (Group A vs. Group B) are compared; these analyses include the following: 1) gene-level associations between DNA methylation changes and gene expression changes (comparison) and 2) visualization of DNA methylation and gene expression data together (heatmap).

### Data preprocessing

The inputs of MethGET are DNA methylation (CGmap file as methylation calls), gene expression (tab-delimited text file), and gene annotation (GTF file) data. The quality control of DNA methylation (WGBS) and gene expression data (RNA-seq) is usually performed before or during alignment. The quality control methods such as FastQC and NGS QC Toolkit in the read alignment step would help provide good inputs for MethGET to improve the accuracy of subsequent analyses [27, 28]. CGmap files including the DNA methylation levels, read counts and methylation context of each cytosine are the output of the bisulfite specific aligners such as BS-Seeker and its variants [29–31]. Other methylation calling files can be converted to CGmap format by MethGET, including CX report files generated by Bismark, the methylation calls generated by methratio.py in BSMAP (v2.73), the allc files by methylpy, and the TSV files exported from the methylation calling status with METHimpute [32–35]. To accelerate the retrieval of methylation information, MethGET converts CGmap data into three contexts (CG, CHG, CHH) in binary compressed format files (bigwig format) [36]. Gene expression values represent quantitative measurements of gene expression. The gene expression input of MethGET is a tab-delimited txt file containing gene names and gene expression values such as RPKM (reads per kilobase per million mapped reads) and FPKM (fragments per kilobase of transcript per million), and CPM (counts per million). The gene annotation GTF file contains gene names and the transcript annotation of the genome available from the Ensembl FTP server (https://asia.ensembl.org/info/data/ftp/index.html). MethGET parses the GTF file into four BED formats for different genomic locations: gene bodies, promoters, exons, and introns. The gene body is defined as the region from the transcription start site (TSS) to the transcription end site (TES), and the promoter is defined as the region two kilobases upstream of the gene body. Finally, MethGET averages the methylation levels at different genomic locations for downstream analysis and methylome visualization. MethGET can also preprocess TE GTF to BED format and allow the correlation between TE methylation and TE expression in the downstream analyses (Additional file 2: Figure S1).

### Single-methylome analyses

Single-methylome analyses investigate the association between the methylome and transcriptome within a single sample. We demonstrate the following single-methylome analyses using the data from human cancer-associated fibroblasts [37] and *Arabidopsis thaliana* ecotype Columbia [38].

#### Correlation analyses of genome-wide DNA methylation and gene expression (correlation)

**Fig. 2:**
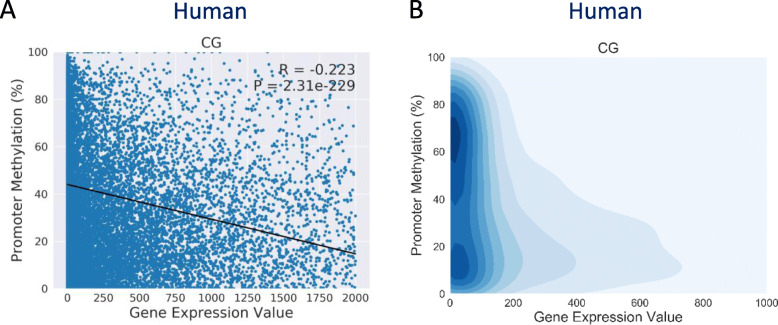
Correlation analyses of genome-wide DNA methylation and gene expression (human data). **a** Scatterplot of promoter methylation levels (y-axis) and gene expression values (x-axis). The correlation coefficient (R) and *p*-value (P) are provided in the top right corner of the plot. **b** The 2D kernel density plot of (**a**)

To display the correlation between genome-wide DNA methylation and gene expression, MethGET generates scatterplots and 2D kernel density plots. The values of Pearson’s and Spearman’s correlation coefficients (R) are provided, as well as the accompanying *p*-values from Student’s t-test. Typically, promoter methylation tends to present a negative correlation (*R* < 0) in which an increased methylation level correlates with decreased gene expression values (Fig. 2a). Since over-plotting often occurs in the scatterplot, a 2D kernel density plot is also provided to represent the density distribution. Groups of genes can be identified on the basis of deeper coloration; for example, it can be seen in Fig. 2b that genes with lower expression are enriched in both high and low DNA methylation levels.

#### Ordinal association analyses with genes ranked by gene expression level (ordinal association)

**Fig. 3:**
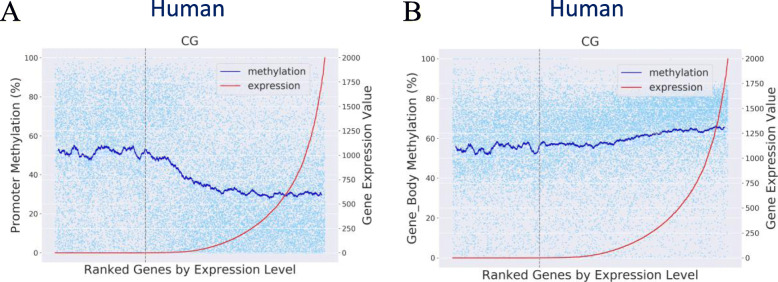
Ordinal association analyses with genes ranked by gene expression level (human data). Scatterplot and fitting curves of DNA methylation and relative gene expression. **a** Promoter methylation and **b** gene body methylation. The grey line in the plot separates genes into unexpressed genes on the left side (gene expression value = 0) and expressed genes on the right side (gene expression value > 0)

To investigate the methylation pattern associated with relative gene expression, MethGET provides scatterplots with genes ranked by gene expression level from low expression levels to high expression levels. Additionally, MethGET can generate fitting curves for the scatterplot via the moving average method to smooth out noise and highlight trends of methylation. In Fig. 3, the promoter methylation trend decreases with increasing gene expression values, but the gene body methylation trend increases slightly with increasing gene expression; suggesting a differential association or usage between DNA methylation and gene expression at different genomic regions.

#### Distribution of DNA methylation by groups of genes with different expression levels (grouping statistics)

**Fig. 4:**
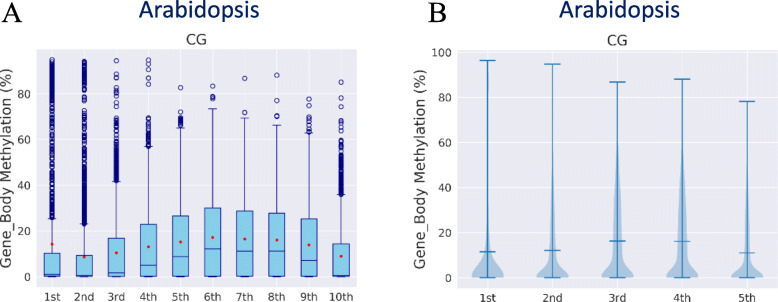
Distribution of DNA methylation by groups of genes with different expression levels (Arabidopsis data). **a** The boxplot shows the gene body methylation pattern in 10 different gene expression groups. **b** Violin plot of (**a**) with five expression groups

To better reveal the complex regulation of methylation, in MethGET both boxplots and violin plots are provided to visualize the central tendency and dispersion of DNA methylation levels according to groups with different gene expression levels (Fig. 4). Genes are grouped as non-expressed genes and 5 quantiles of expressed genes according to the gene expression level groups from low to high; the 1st quintile is the lowest, and the 5th is the highest. In addition, the correlation coefficient of DNA methylation and gene expression in each group as well as descriptive statistics (such as the mean and standard deviation) are available in the provided spreadsheet (Additional file 2: Table S1).

#### Average methylation level profiling according to different expression groups around genes (metagene)

**Fig. 5:**
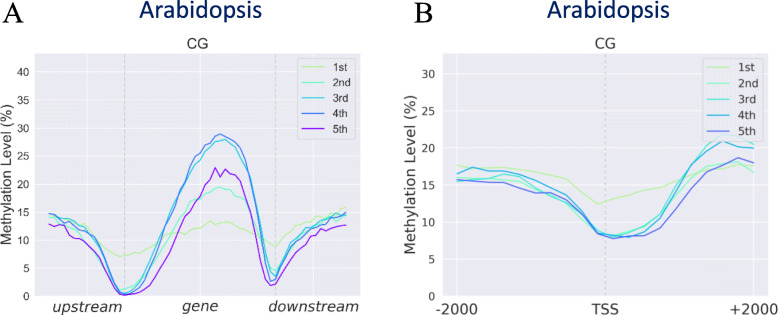
Average methylation level profiling according to different expression groups around genes (Arabidopsis data). **a** The “region” plot shows the DNA methylation pattern around the gene body region. **b** The “site” plot shows the methylation pattern around the transcription start site (TSS)

To profile DNA methylation around genes across different expression groups, MethGET provides two kinds of metagene plots: “region” and “site” plots (Fig. 5). For a “region” plot, gene body regions are divided into 30 windows based on the region’s length, and the average methylation level is calculated for each window. The methylation patterns both upstream and downstream of genes are shown for half of the gene body (i.e., 15 windows). On the other hand, a “site” plot allows the methylation adjacent to a specific reference point (transcription start site or transcription end site) to be visualised. This can help to elucidate the mechanisms of DNA methylation at certain bases around a specific point. The regions two kilobases upstream and downstream of the reference point are divided into 10 windows, and the average methylation level is calculated in each window. A single-base resolution is possible in a “site” plot when the number of windows is equal to the number of bases. In this analysis, users can define the number of groups for separating genes by gene expression levels, and they can also define the number of windows in “region” and “site” plots for averaging DNA methylation levels.

### Multiple-methylome analyses

Multiple-methylome analyses investigate the correlation between alterations in methylomes and the differences in transcriptomes between two groups of samples (e.g., mutant vs. wild type or cancer vs. normal). Moreover, the correlation can be explored at the gene level to understand the DNA methylation regulatory network associated with gene expression changes. To demonstrate the multiple-methylome analysis process, we applied MethGET to the *otu5* mutant (Group A) and wild type (Group B) of Arabidopsis (GEO accession: GSE81407) [38].

#### Gene-level associations between DNA methylation changes and gene expression changes (comparison)

**Fig. 6:**
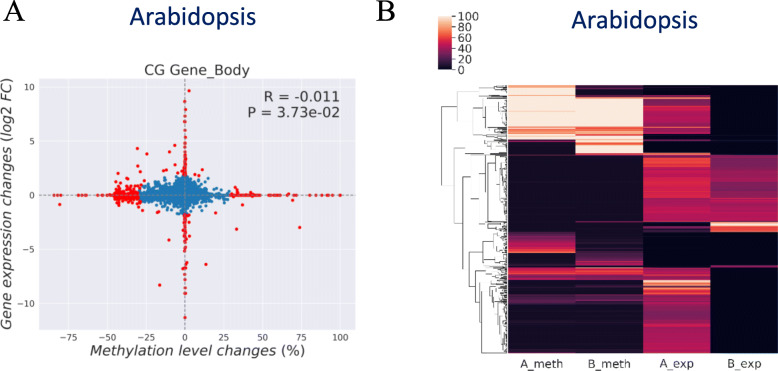
Multiple-methylome analyses (Arabidopsis mutant (Group **a**) vs. wild type (Group **b**)). **a** Gene-level associations between DNA methylation changes and gene expression changes. The red dots represent differential genes of DNA methylation and gene expression (bi-variate Gaussian mixture model; *p*-value < 10^− 6^). **b** Visualization of DNA methylation and gene expression data together

DNA methylation changes between two groups of samples may exert a specific functional impact on gene expression between them (e.g., mutants, treatments, stresses). To calculate the changes between two groups (Group A vs. Group B), MethGET first averages DNA methylation levels and gene expression within an individual group. The correlation between methylation level changes (Group A – Group B) and gene expression changes (log2 (Group A/Group B)) can be shown throughout the genome (Fig. 6a). The overall correlation can be measured by using Pearson’s correlation coefficient and the accompanying *p*-value.

To identify the genes with clear changes of DNA methylation and gene expression (i.e., differential genes), we incorporated the Gaussian Mixture Model (GaussianMixture module from the scikit-learn package in python) in the bi-variate correlation plot [39]. As the default setting, a data point will be defined as differential genes if *p*-value < 10^− 6^ in GMM. They are marked in red color in the scatterplot, and the users can choose to show the number of differential genes in the four quadrants of the plots. These genes with different DNA methylation statuses associated with gene expression changes are important because their expression may potentially be regulated by differences in DNA methylation between the two groups. The information for the differential genes (gene names, methylation levels, and gene expression values) in the output table allows for downstream analyses such as KEGG pathway analysis or Gene Ontology functional analysis [40, 41].

#### Visualization of DNA methylation and gene expression data together (heatmap)

MethGET provides a heatmap representation for the visualisation of both WGBS data and RNA-seq data between two groups (Fig. 6b). Each row represents a gene, and the DNA methylation level and gene expression are averaged within each group in the columns. Hierarchical clustering of similar methylation and gene expression patterns can also be performed, and the resulting dendrogram is presented at the left margin of the heatmap. This is useful for identifying genes that are commonly regulated, and the order of the clustered genes will be listed in the output table.

## Results and discussion

MethGET is available through both the web application and the stand-alone version for command-line usage. On the web platform, users can directly upload their datasets and download all output figures with a high resolution of 300 dpi in one click. In the stand-alone version, MethGET can be executed in a local Unix/Linux environment. The web tutorial is provided in Additional file 1, and guidance regarding the stand-alone version is provided at the GitHub repository. MethGET also provides example Arabidopsis data for users to explore the tool’s functions. We evaluated the performance of MethGET on the Intel Xeon E5–2650 processor (384GB RAM; clock speed 2.0GHz). The processing time with and without metagene analyses for Arabidopsis, rice, human, and wheat are in Table 1. The processing time is not ultra-fast and will be multiplied by the number of samples. MethGET can cover most genomes from Arabidopsis (135 Mb), rice (350 Mb), human (3.2 Gb) to Wheat (14.5 Gb). The processing time without metagene analyses for smaller genomes such as Arabidopsis (135 MB) can be available in approximately 30 min. After processing, the figures are available within minutes.

**Table 1:** The processing time of Arabidopsis, human, rice, and wheat in MethGET

	Arabidopsis	Rice	Human	Wheat
Genome size (Mb)	135	380	3200	14,500
Gene number	27,655	39,045	20,805	107,891
Processing time without metagene analyses (hrs:mins:secs)	00:32:51	01:20:42	03:47:11	06:38:14
Processing time with metagene analyses (hrs:mins:secs)	04:21:15	07:50:36	09:47:52	18:31:14

The tests are on Intel Xeon E5–2650 processor (384GB RAM; clock speed 2.0GHz)

### Demonstration of MethGET with rice data

**Fig. 7:**
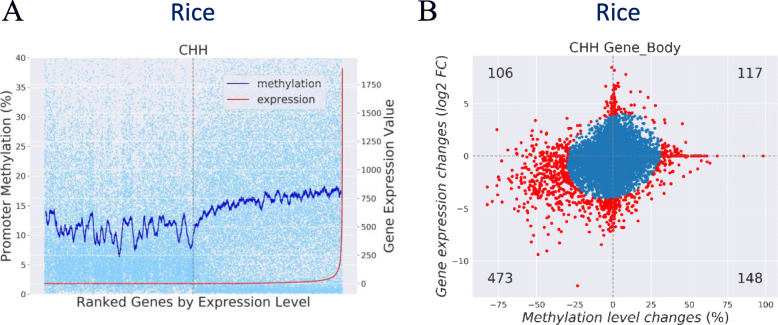
Demonstration of the application of MethGET with rice data. **a** The CHH methylation level trend increased with the increase in gene expression in the promoter region. **b** Most differential genes showed decreases in both CHH methylation and gene expression in gene body regions. The number of differential genes is shown in the four quadrants of the plot

To test the utility of MethGET for other species, we downloaded Japonica rice data (cv. TNG67) from the embryonic stage and successfully regenerated calli (GEO accession: GSE82138) [26]. We first investigated the relationship between DNA methylation and gene expression in the rice methylome via single-methylome analyses. In the ordinal association analyses presented in Fig. 7a, the CHH methylation level at the promoter region was found to increase with the gene expression. This result is in line with a recent study showing a positive correlation between CHH promoter methylation and gene expression in rice [42].

In addition, we utilized MethGET to examine whether the gene expression changes observed during the tissue culture process were associated with DNA methylation. We conducted multiple-methylome analyses to compare the embryonic stage with successfully regenerated calli in rice (regenerated callus vs. embryonic stage). Figure 7b shows that most genes showing a significantly changes of the CHH gene body methylation and gene expression (bi-variate Gaussian mixture model; *p*-value < 10^− 6^) are enriched in the third quadrant. This demonstrated that the embryonic stage is characterized by lower methylation levels and lower gene expression compared to the regenerated calli. The results suggested that most genes exhibit decreases in both CHH methylation and gene expression in gene body regions as the embryo develops into a regenerated callus, which was not reported in the original paper [26]. We further performed the analyses again with non-TE-related genes and found that the results still hold in the absence of TE-related genes (Additional file 2: Figure S2). Therefore, MethGET is not only a useful tool for investigating the effects of DNA methylation on gene expression but also able to reveal novel results.

### Feature comparison with other bioinformatic software

We compared MethGET with different methylation pipelines (COHCAP, PiiL, and ViewBS) and methylation databases (MethHC, and iMETHYL), and their features are listed in Table 2. Only a few tools can integrate DNA methylation with gene expression. COHCAP is an integrative pipeline for CG methylation data produced either from an Illumina methylation array or by targeted bisulfite sequencing. It provides differential methylation analyses and the correlations between DNA methylation and gene expression [21]. The PiiL tool is an integrated DNA methylation and gene expression pathway browser. It allows the visualization of CpG methylation and gene expression related to pathways in a single sample or groups of samples [22]. Both COHCAP and PiiL are restricted to the analysis of CG methylation. In addition, for the annotation of a species, COHCAP users need to create custom CpG island annotations for the species on the basis of targeted bisulfite sequencing data, and PiiL can only be applied to organisms in which pathway and network data are available. ViewBS is a toolkit for visualizing bisulfite sequencing data [23]. For correlating between DNA methylation and gene expression, ViewBS provides metagene plots in the MethOverRegion function. ViewBS requires users to provide different gene region files that are separated and sorted by gene expression level in advance. The method is not designed for genome wide correlation and the users need to process the expression data first.

Several methylation databases can be used to visualize DNA methylation and gene expression on a web platform. MethHC is a database of DNA methylation and mRNA/microRNA expression data from 18 human cancers from TCGA (The Cancer Genome Atlas). It provides a web-based interface for the profiling of methylation patterns in normal and tumour tissues and the analysis of the correlation between the methylation and expression of genes related to certain cancers [24]. iMETHYL is an integrative database of human DNA methylation, gene expression, and genomic variation data. It provides cell-type-specific browser tracks (e.g., CD4T, monocytes, and neutrophils) for examining DNA methylation variation, gene expression and single-nucleotide variants in any region of the human genome [25]. These tools are convenient because they provide an interface for representing the results of integrative analyses of methylation and expression data directly from databases. However, they do not allow the analysis of user-provided data and are limited to CG methylation in the human genome.

MethGET provides comprehensive analyses correlating WGBS data and RNA-seq data. It is the only tool that correlates gene expression with CG, CHG, and CHH methylation. Furthermore, it has a user-friendly web interface for customized analyses of all species for which annotations are available. We believe that researchers can easily use MethGET to investigate the epigenetic regulation of gene expression by DNA methylation.

**Table 2:** Feature comparison of MethGET with other tools

	MethGET	COHCAP	PiiL	ViewBS	MethHC	iMETHYL
Inputs with methylation and expression data	○	○	○	X (Using gene region files with different expression values)	No input	No input
Customized analyses	○	○	○	○	X	X
Web interface	○	X	X	X	○	○
Multiple-methylome analyses	○	○	○	○	○	X
Supported species	Any species with annotated genome	Species with CpG island annotation	Species with pathway annotation	Species with region file	*Homo sapiens*	*Homo sapiens*
Sequence context of cytosine methylation	CG, CHG, CHH	CG	CG	CG, CHG, CHH	CG	CG
Target regions	Genome-wide	Selected CpG islands	Genes in the pathway	Genome-wide	Selected genes	Selected genes or regions
Genomic locations	Gene body, promoter, exon, intron, TE	CpG islands	Annotated CpG sites	Regions in region files	8 Gene regions+, 5 CpG Island regions	Only selected cytosines
Correlation analyses	6 analyses	1 (Scatterplot)	Visualization	1 (Metagene plot)	1 (Scatterplot)	Visualization
Citation	Teng et al. (2020)	Warden et al. (2013) [21]	Moghadam et al. (2017) [22]	Huang et al. (2018) [23]	Huang et al. (2015) [24]	Komaki et al. (2018) [25]

## Conclusions

MethGET was developed for the correlation of genome-wide DNA methylation and gene expression data. The comprehensive analyses that can be performed at the web interface provide customized analyses that allow users to explore epigenetic regulation in an efficient way. The address of the MethGET website is https://paoyang.ipmb.sinica.edu.tw/Software.html, and a step-by-step manual is provided in Additional file 1. Guidance regarding the use of MethGET and its module requirements can be found at Github (https://github.com/Jason-Teng/MethGET).

## Availability and requirements

Project Name: MethGET

Project Home Page: https://paoyang.ipmb.sinica.edu.tw/Software.html

Operating system: Platform independent.

Programming Language: Python/Django.

Other requirements: web browsers, internet connectivity

License: None

Any restrictions to use by non-academics: None

## Supplementary information

**Additional file 1.** Step-by-step tutorial of the MethGET web interface.

**Additional file 2: Table S1.** The output spreadsheet of Fig. 4b from grouping statistics. **Figure S1.** The correlation between TE methylation and TE expression from MethGET (Arabidopsis). **Figure S2.** The correlation between genic CHH methylation and gene expression in all genes and non-TE-related genes.

## Data Availability

All datasets used in this study have been previously published. The Arabidopsis and human data are available in the Gene Expression Omnibus repository [https://www.ncbi.nlm.nih.gov] under GEO accession GSE81407 and GSE86260. The rice data are available from GEO accession GSE82138.

## Abbreviations

NGS: Next-generation sequencing
WGBS: Whole-genome bisulfite sequencing
RNA-seq: RNA-sequencing
RPKM: Reads per kilobase per million mapped reads
FPKM: Fragments per kilobase of transcript per million
CPM: Counts per million
TSS: Transcription start site
TES: Transcription end site
TCGA: The Cancer Genome Atlas
