# Supplementary material for: MethGET: web-based bioinformatics software for correlating genome-wide DNA methylation and gene expression
Source: BMC Genomics. 2020 May 29;21:375. doi: 10.1186/s12864-020-6722-x (PMC7257144; doi:10.1186/s12864-020-6722-x)

Tutorial of MethGET website

The web-based interface helps MethGET to conduct analyses in a user-friendly and efficient way. Users with no programming skills and command-line experience can enjoy the analyses. Here is the tutorial for step-by-step instruction to guide the users to go through the process.

Overview

The whole process can be simply divided into three steps.

I. Upload data.
II. Sign in and launch analyses.
III. Get results.

After uploading data, it will take time to preprocess the data (0.5hr in one sample and 4.0hr for one metagene in Arabidopsis). Once the preprocess finished, you would receive an "Account name" from the email. Then you can sign in the account name and go to the analyses tab. Choose the parameters and click "Submit" to launch analyses. The results will show on the screen within minutes and you can download all results with one click.

I. Upload data

Click the "Upload and sign in" tab in the navigation menu and follow the instructions below.

Step 1: Enter Your Project Name and Email.
Giving the name of this project to avoid confusion when you have many projects. Your email name needs to be valid because we will give you the account name for analyses after preprocessing.

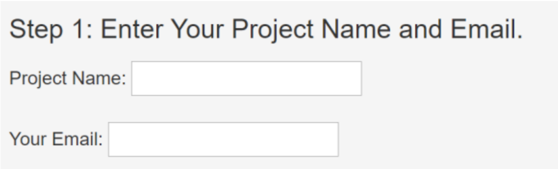

Step 2: Upload DNA Methylation (CGmap.gz) and Gene Expression Files (Exp.txt).
Upload a sample for single methylome analyses. Check the checkbox of the sample and upload DNA methylation and gene expression files. It will take 30 minutes for Arabidopsis data.

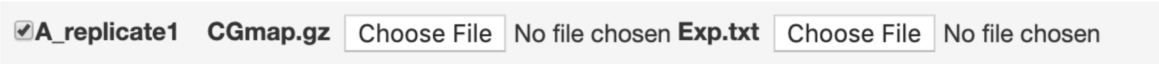

Metagene analyses need to check certain checkbox. The default takes 3.5hr for Arabidopsis data. Other parameters for metagene can be chosen by clicking the "+" button.

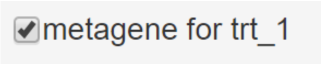

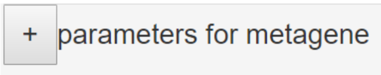

Multiple methylome analyses require at least one sample from the treatment group and one sample from the control group.

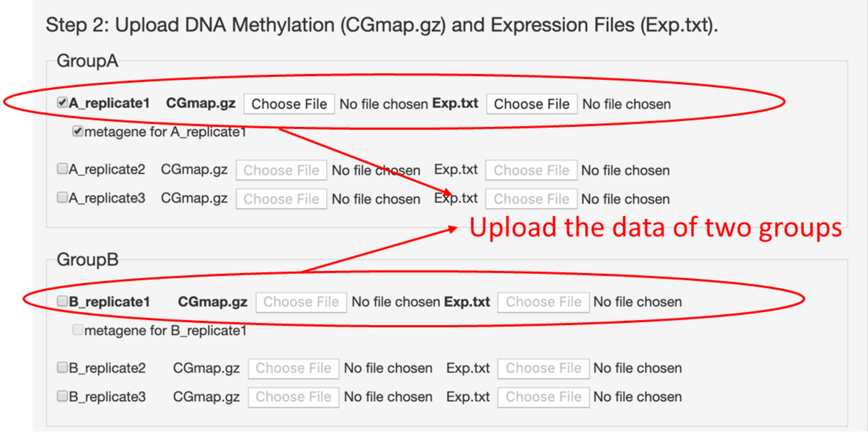

Sample data can be accessed by users from the button “Download Example Data” so that the users can confirm CGmap.gz and Exp.txt format. Please make sure the format is correct for analyses.

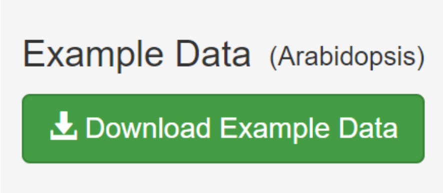

Step 3: Choose The Organism.

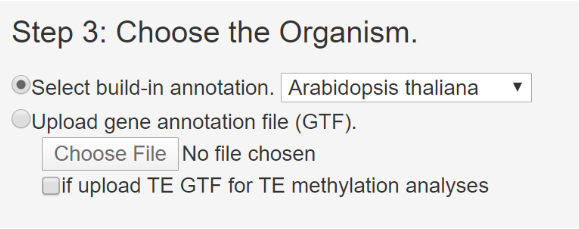

We have built in the annotation of five species including Arabidopsis, human (hg19), mouse (mm10), rice, and zebrafish. The users can select them without uploading the annotation file. You can also upload the customized GTF file for other species. The users can upload the TE GTF file when checking the checkbox "if upload TE GTF for TE methylation analyses". In this way, the expression inputs are also needed to be TE expression.

Step 4: Click the “submit” button.

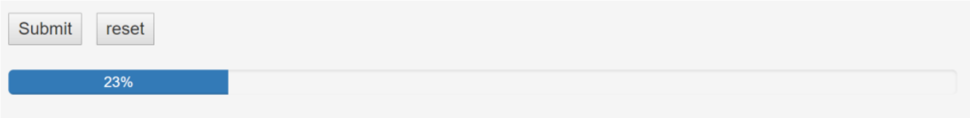

After clicking “submit”, your files are uploading to the webserver and the progress bar shows the process of uploading data. Once successfully upload the data to the web, the alert box will display then you can close

the browser. You will receive an email to make sure we received the data and your email is valid. Then we have started the preprocessing, please wait for the email and account name when we finished preprocessing.

Ps: It is better to upload the files under a good network because the data is usually large.

II. Launch analysis

Step 1: Sign in the account name

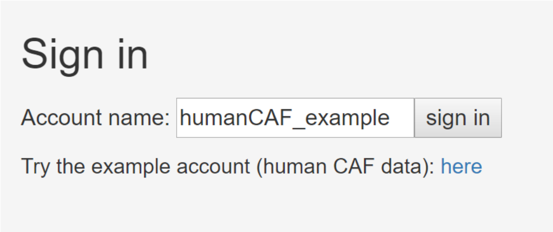

When the preprocess finished, users will receive an account name in the email. Click the “Upload and sign in” tab in the navigation menu. Paste the account name copied from email and click the “sign in” button. The alert box will show if you successfully sign in or not.

Ps: Try the example account by clicking “here” and click sign in. The example account contains human data with treatment: cancer-associated fibroblasts (CAFs), and control: normal prostate fibroblasts (NPFs).

Step 2: Choose the analyses and set the parameters

Pull down the navigation bar and click the name of the analyses. Choose the sample and other parameters on the left side.

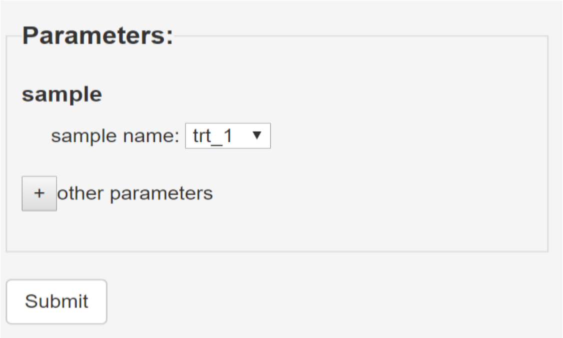

Step 3: Click the “submit” button.

Click the “Submit” button. Wait within minutes and the results will be displayed on the right-hand side.

III. Get results

Once the results showing up on the right-hand side, you can click the “CG”, “CHG”, and “CHH” button to view the results in different methylation contexts.

Click “download all” to download all results with different methylation contexts (CG, CHG, CHH) and target regions (promoter, gene body, exon, intron). You can also download selected figures and tables from “download figure’’ and “download table”.

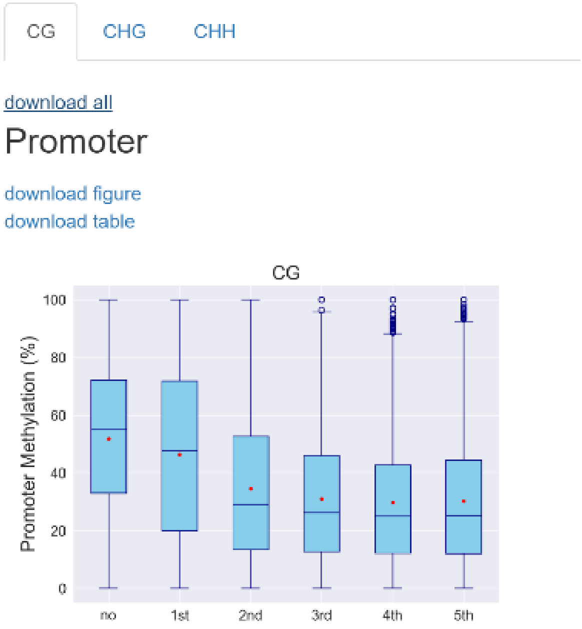

Supplement: Supplementary file 1 — Additional file 1. Step-by-step tutorial of the MethGET web interface. [file 12864_2020_6722_MOESM1_ESM.pdf]
