# Supplementary material for: MethGET: web-based bioinformatics software for correlating genome-wide DNA methylation and gene expression
Source: BMC Genomics. 2020 May 29;21:375. doi: 10.1186/s12864-020-6722-x (PMC7257144; doi:10.1186/s12864-020-6722-x)
Supplement: Supplementary file 2 — Additional file 2: Table S1. The output spreadsheet of Fig. 4b from grouping statistics. Figure S1. The correlation between TE methylation and TE expression from MethGET (Arabidopsis). Figure S2. The correlation between genic CHH methylation and gene expression in all genes and non-TE-related genes. [file 12864_2020_6722_MOESM2_ESM.pptx]

## Slide 1
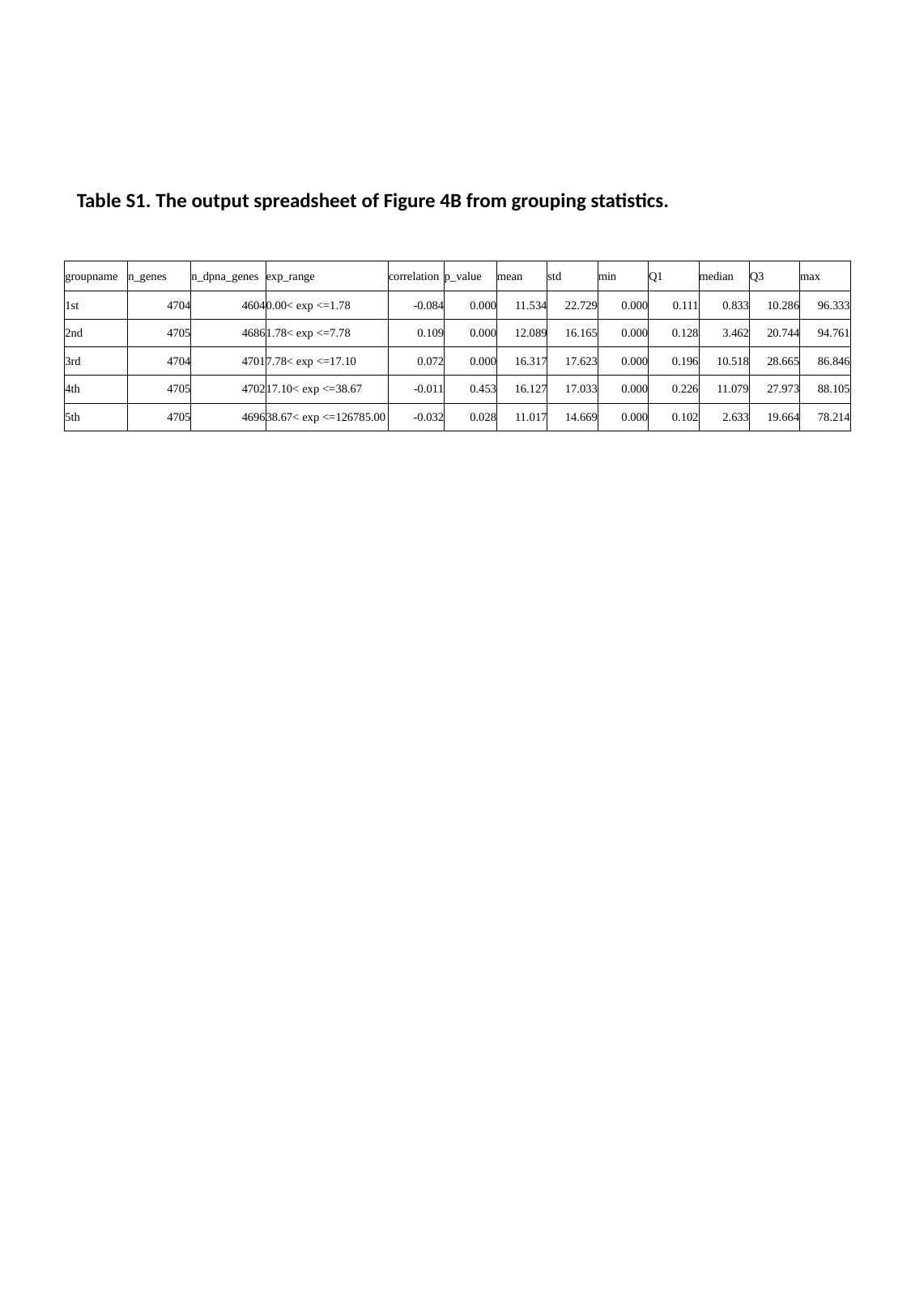

Table S1. The output spreadsheet of Figure 4B from grouping statistics.
| groupname | n\_genes | n\_dpna\_genes | exp\_range | correlation | p\_value | mean | std | min | Q1 | median | Q3 | max |
| --- | --- | --- | --- | --- | --- | --- | --- | --- | --- | --- | --- | --- |
| 1st | 4704 | 4604 | 0.00< exp <=1.78 | -0.084 | 0.000 | 11.534 | 22.729 | 0.000 | 0.111 | 0.833 | 10.286 | 96.333 |
| 2nd | 4705 | 4686 | 1.78< exp <=7.78 | 0.109 | 0.000 | 12.089 | 16.165 | 0.000 | 0.128 | 3.462 | 20.744 | 94.761 |
| 3rd | 4704 | 4701 | 7.78< exp <=17.10 | 0.072 | 0.000 | 16.317 | 17.623 | 0.000 | 0.196 | 10.518 | 28.665 | 86.846 |
| 4th | 4705 | 4702 | 17.10< exp <=38.67 | -0.011 | 0.453 | 16.127 | 17.033 | 0.000 | 0.226 | 11.079 | 27.973 | 88.105 |
| 5th | 4705 | 4696 | 38.67< exp <=126785.00 | -0.032 | 0.028 | 11.017 | 14.669 | 0.000 | 0.102 | 2.633 | 19.664 | 78.214 |

## Slide 2
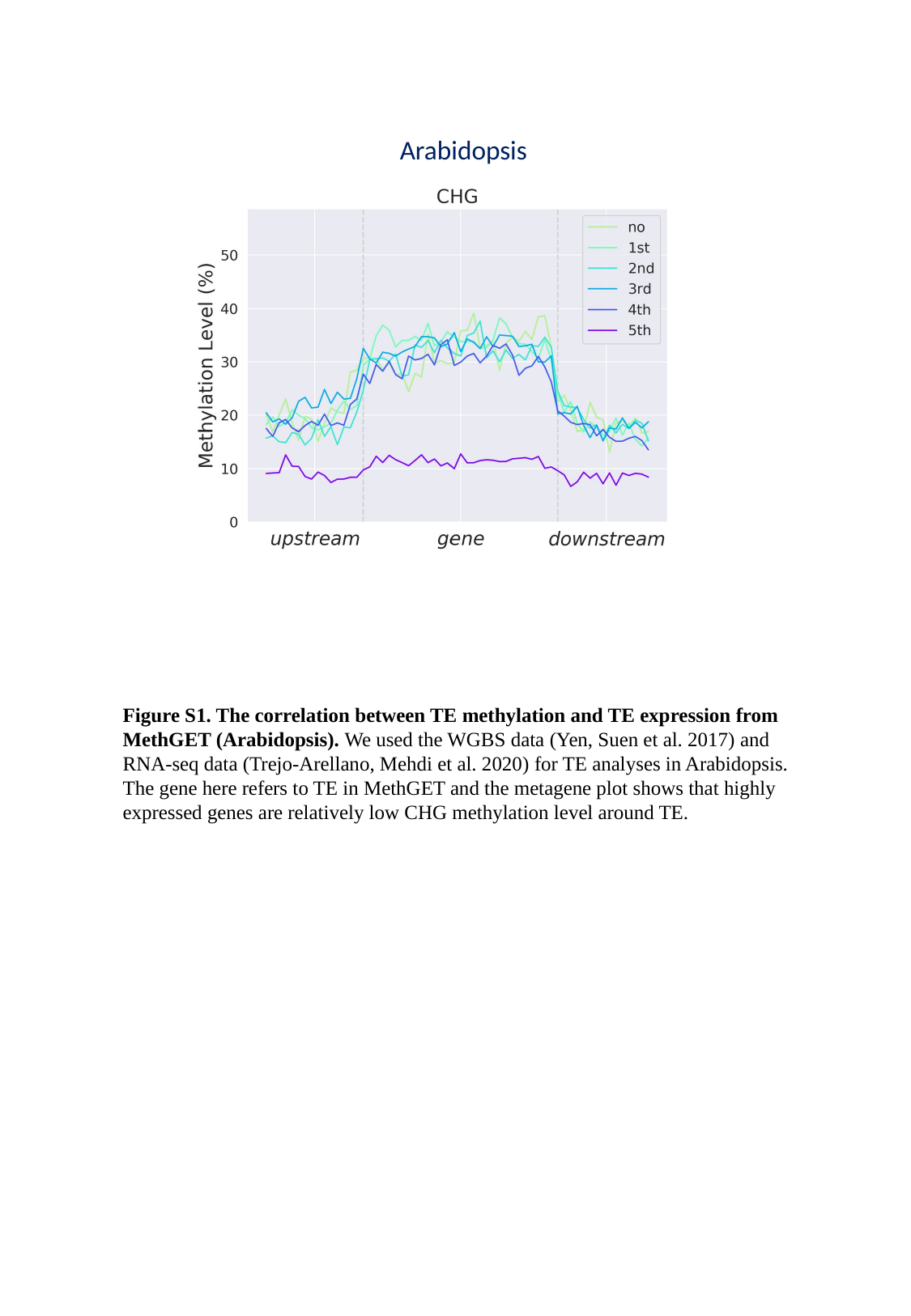

Arabidopsis
Figure S1. The correlation between TE methylation and TE expression from MethGET (Arabidopsis). We used the WGBS data (Yen, Suen et al. 2017) and RNA-seq data (Trejo-Arellano, Mehdi et al. 2020) for TE analyses in Arabidopsis. The gene here refers to TE in MethGET and the metagene plot shows that highly expressed genes are relatively low CHG methylation level around TE.

## Slide 3
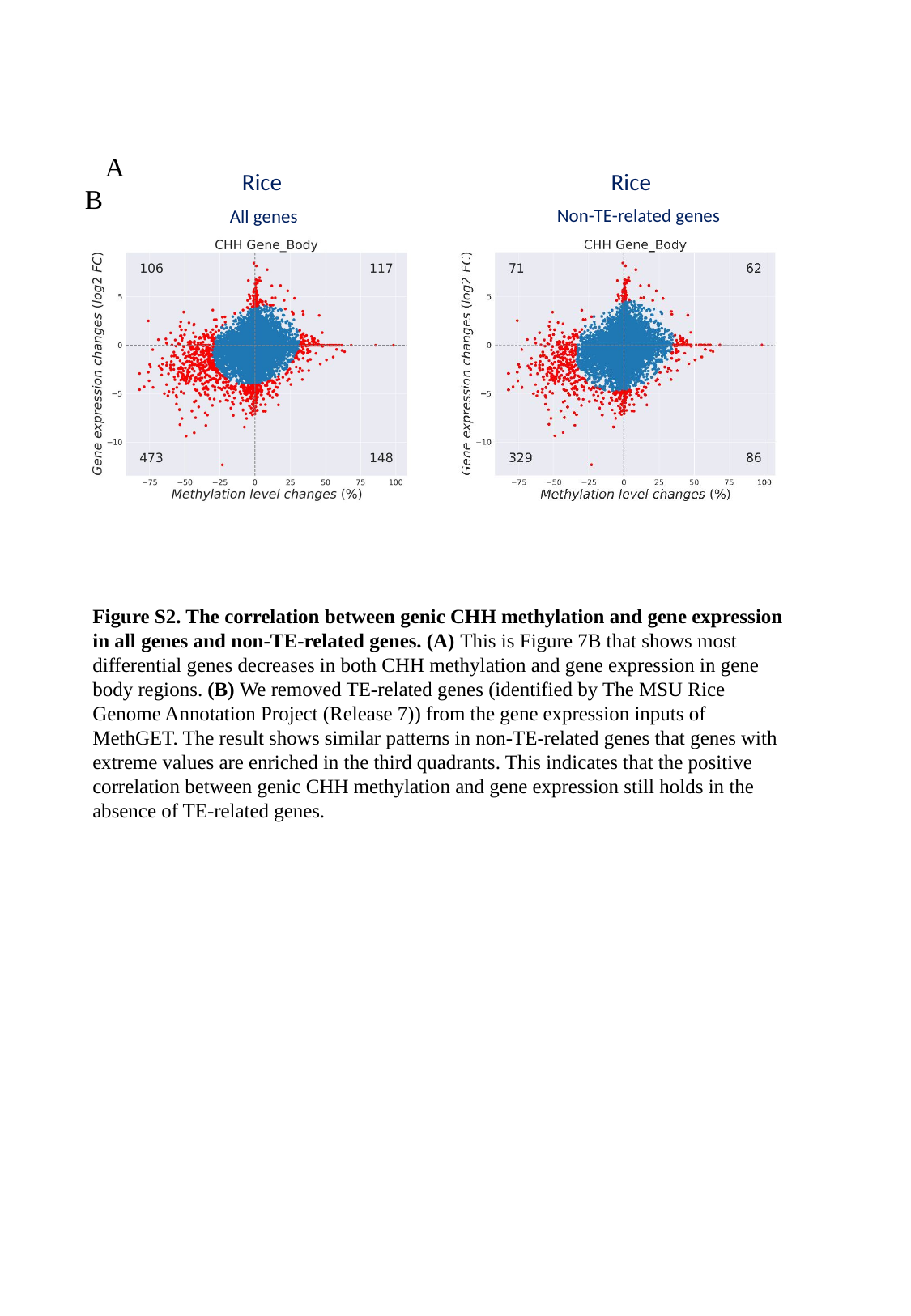

A B
Rice
Rice
Non-TE-related genes
All genes
Figure S2. The correlation between genic CHH methylation and gene expression in all genes and non-TE-related genes. (A) This is Figure 7B that shows most differential genes decreases in both CHH methylation and gene expression in gene body regions. (B) We removed TE-related genes (identified by The MSU Rice Genome Annotation Project (Release 7)) from the gene expression inputs of MethGET. The result shows similar patterns in non-TE-related genes that genes with extreme values are enriched in the third quadrants. This indicates that the positive correlation between genic CHH methylation and gene expression still holds in the absence of TE-related genes.
